# Evolution and Phenotypic Selection of Cancer Stem Cells

**DOI:** 10.1371/journal.pcbi.1004025

**Published:** 2015-03-05

**Authors:** Jan Poleszczuk, Philip Hahnfeldt, Heiko Enderling

**Affiliations:** 1 Center of Cancer Systems Biology, GRI, Tufts University School of Medicine, Boston, Massachusetts, United States of America; 2 College of Inter-faculty Individual Studies in Mathematics and Natural Sciences, University of Warsaw, Warsaw, Poland; University of Chicago, UNITED STATES

## Abstract

Cells of different organs at different ages have an intrinsic set of kinetics that dictates their behavior. Transformation into cancer cells will inherit these kinetics that determine initial cell and tumor population progression dynamics. Subject to genetic mutation and epigenetic alterations, cancer cell kinetics can change, and favorable alterations that increase cellular fitness will manifest themselves and accelerate tumor progression. We set out to investigate the emerging intratumoral heterogeneity and to determine the evolutionary trajectories of the combination of cell-intrinsic kinetics that yield aggressive tumor growth. We develop a cellular automaton model that tracks the temporal evolution of the malignant subpopulation of so-called cancer stem cells(CSC), as these cells are exclusively able to initiate and sustain tumors. We explore orthogonal cell traits, including cell migration to facilitate invasion, spontaneous cell death due to genetic drift after accumulation of irreversible deleterious mutations, symmetric cancer stem cell division that increases the cancer stem cell pool, and telomere length and erosion as a mitotic counter for inherited non-stem cancer cell proliferation potential. Our study suggests that cell proliferation potential is the strongest modulator of tumor growth. Early increase in proliferation potential yields larger populations of non-stem cancer cells(CC) that compete with CSC and thus inhibit CSC division while a reduction in proliferation potential loosens such inhibition and facilitates frequent CSC division. The sub-population of cancer stem cells in itself becomes highly heterogeneous dictating population level dynamics that vary from long-term dormancy to aggressive progression. Our study suggests that the clonal diversity that is captured in single tumor biopsy samples represents only a small proportion of the total number of phenotypes.

## Author Summary

We present an *in silico* computational model of tumor growth and evolution according to the cancer stem cell hypothesis. Inheritable traits of cells may be genetically or epigenetically altered, and traits that confer increased fitness to the cell will be selected for on the population level. Phenotypic evolution yields aggressive tumors with large heterogeneity, prompting the notion that the cancer stem cell population per se is highly heterogeneous. Within aggressive tumors cancer stem cells with low tumorigenic potential may be isolated. Simulations of our model suggest that the cells harvested in core needle biopsies represent less than 10% of the phenotypic heterogeneity of the total tumor population. Dependent on the cells captured in the sample, xenografted tumors may exhibit aggressive growth or long-term dormancy—dynamics that may suggest opposing treatment approaches for the same tumor when translated into clinical decision-making.

## Introduction

Human organs and tissues are comprised of cells that have evolved to maintain functionality and integrity. Cells of different organs and ages have different traits, such as migration rate, turnover time, proliferation potential and lifespan until senescence. While fetal diploid cell strains in culture demonstrate a large number of divisions before mitotic arrest and culture degeneration (50 ± 10 population doublings, termed the “Hayflick Limit” [1–3]), hematopoietic progenitors may undergo about 20–30 divisions [4], and colonic crypt progenitors complete only four to six divisions before getting washed off at the top of the crypt [3]. Repopulation of the tissue is assured by tissue stem cells that sit on top of a cellular hierarchy [5,6]. In a physiological setting, stem cells are predominantly non-mitotic to prevent malignant transformation [7] and only enter the mitotic cycle when tissue repopulation is required [8–10]. The transit-amplifying offspring of a stem cell may undergo multiple divisions to produce a population of cells that differentiate into tissue-specific cells with determined function and lifespan. The potencies of tissue stem cells and transit-amplifying cells vary with tissue type and age. Transformation may occur at any time in all tissue compartments, but the ability of transformed cells to initiate and sustain pathologic tumor growth requires certain kinetic properties including longevity, migration potential, self-renewal and differentiation capacity. These traits are comparable to physiologic stem cells, and cancer cells with such properties have been termed cancer stem cells causing a long and active discussion about the cell of origin of tumor [5,11,12]. Intestinal cancer may be initiated by a transformed stem cell [13], but a transformed progenitor cell with acquired stem-like traits is more likely in myeloid malignancies and NF1- and PDGF-driven glioblastoma [14,15]. The set of kinetics in the initial cancer stem cell, however, is initially close to that of the untransformed cell. The kinetics will be inherited by the descendent cells yielding tumor population dynamics ranging from microscopic dormancy to aggressive tumor growth [16]. It is conceivable that the variation in kinetics of cells in different positions of the tissue hierarchy and at different ages gives rise to many trait combinations unfavorable for progression [17]. Inferior cancer cell trait combinations could, at least in part, explain the increasing observations of pathologic but non-advancing lesions [18,19]. Trait fitness, mutation and evolution may augment our understanding why tumors may be more prevalent in certain organs at a specific age range [20].

Regardless of kinetics at time of transformation, cells are subject to mutations, which enables evolution of and selection for more aggressive traits. For example, during mitosis the daughter cells inherit telomeres, the non-coding replicative protective ends of the DNA [21]. Telomeres get shortened during mitosis [22], which offers a quantitative visualization of the remaining cellular proliferation potential [22,23]. Abnormally increased or decreased telomerase activity [24] in cancer stem cells lengthens or shortens telomeric DNA that defines the number of cell divisions for non-stem cancer cell progeny [25,26].

## Results

We first compare growth of the tumor without (control) and with trait mutations. We then perform detailed analysis of the phenotypes that emerged in the smallest, an average-sized, and the largest tumor in the simulated time frame. Using the phenotypic structure of the largest tumor we investigate the population heterogeneity that is represented in needle biopsies.

### Spontaneous undirected mutations promote tumor growth

**Fig 1:**
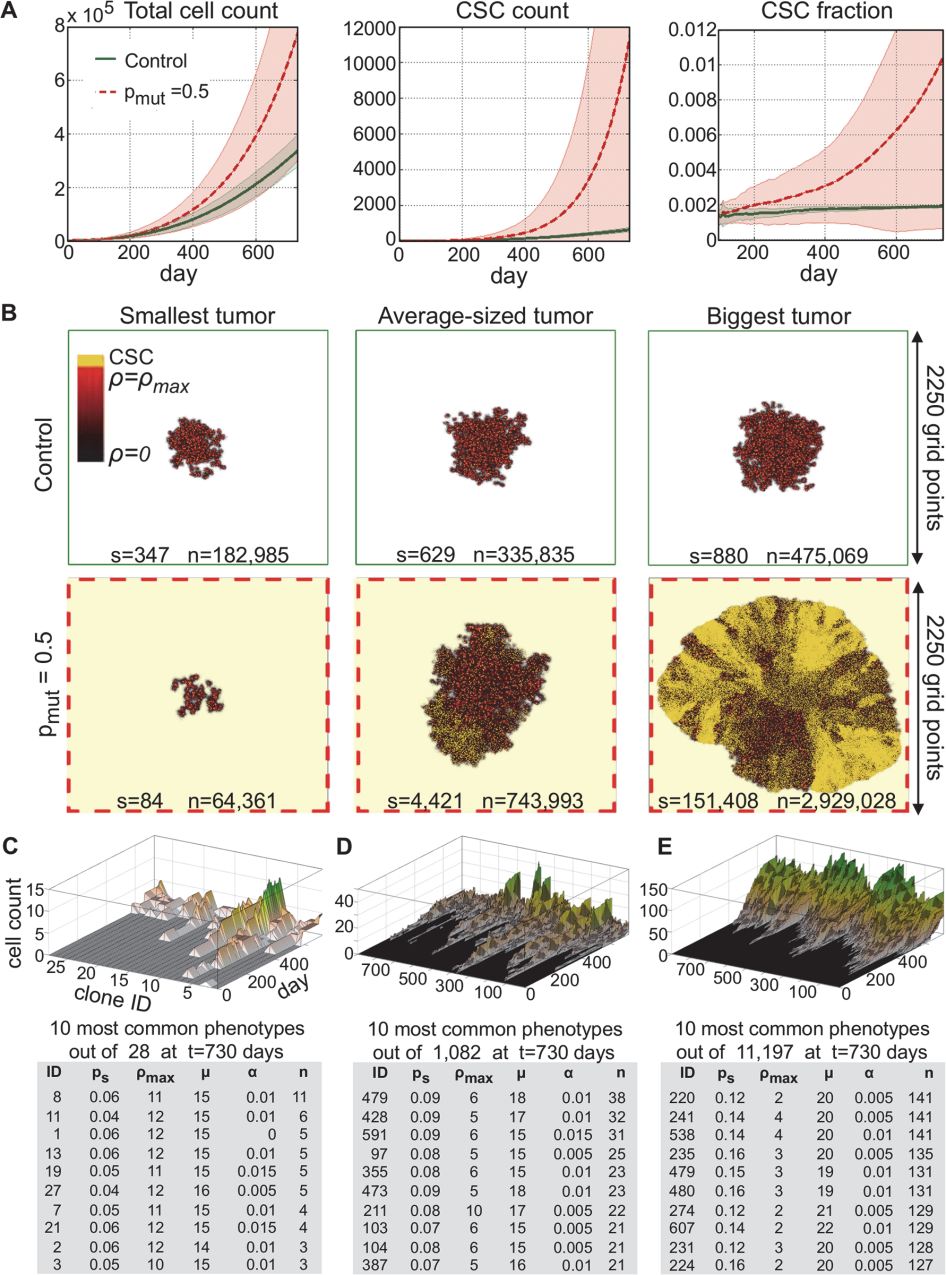
A) Tumor growth curves, cancer stem cell (CSC) number and fraction as a function of time for tumors without evolution (control, green solid curve) and with evolution probability of 50% (p_mut_ = 0.5, red dashed curve). **B)** Simulation snapshots of the smallest, an average-sized and biggest tumor in the control and evolved tumor at t = 730 days. s: number of cancer stem cells; n: number of total cancer cells. **C)** Temporal evolution of the 28 phenotypes in the smallest evolved tumor and trait details of the 10 most common phenotypes. **D)** Temporal evolution of the 1000 most common phenotypes at t = 730 days in an evolved averaged-sized tumor and trait details of the 10 most common phenotypes. **E)** Temporal evolution of the 1000 most common phenotypes at t = 730 days in the biggest evolved tumor and trait details of the 10 most common phenotypes.

We initialize tumor growth simulations with one CSC with the initial traits probability of symmetric division p_s_ = 0.05, proliferation capacity ρ = 10, migration potential μ = 15 and probability of spontaneous death α = 0.01, which has previously been shown to simulate fast tumor growth [16,27]. Motivation for and variation of the discussed parameters α, μ, ρ_max_ and p_s_ and their impact on tumor progression has been discussed in detail elsewhere [16,27,28]. We allow for mutation during symmetric CSC division with a probability of 50% (p_mut_ = 0.5). We simulate tumor growth for t = 730 days, i.e. 2 years, without (control) and with trait evolution. With α = 0.01 and p_s_ = 0.05, stochastic death of the initial CSC before symmetric division and thus regression of the total tumor population is expected and observed in about 20% of the simulations (n = 23). Simulations with successful tumor growth (n = 77) reveal a widespread of tumor sizes after t = 730 days with mutation (standard deviation s.d.>60% of the mean) compared to non-mutating control (s.d.<20% of the mean). Initial growth kinetics (t<200 days) are comparable between both groups, dictated by the chosen initial traits vector. Early tumor growth follows self-metastatic progression as previously described [27,29]. A favorable impact of trait mutations on tumor growth can be seen in significantly steeper growth after selection shapes the population (Fig. 1A). Tumors subject to mutations contain on average approximately 220% more cells with almost a 17-fold increase in CSCs compared to control tumors after t = 730 days. The fastest-growing evolving tumor, however, is more than 6 times larger with 170 times more CSCs than the largest non-evolving control tumor (Fig. 1B). The enrichment in CSC yields a more compact, uniform tumor expansion compared to self-metastatic progression. Evolution, however, may also impair tumor progression. Early mutations that yield unfavorable CSCs kinetics dwarf tumor growth. In the least favorable case, the smallest tumor is only a third the size of the smallest non-evolving control tumor and less than a quarter of the control cancer stem cells (Fig. 1).

### Proliferation potential determines cancer stem cell evolution and tumor growth

**Fig 2:**
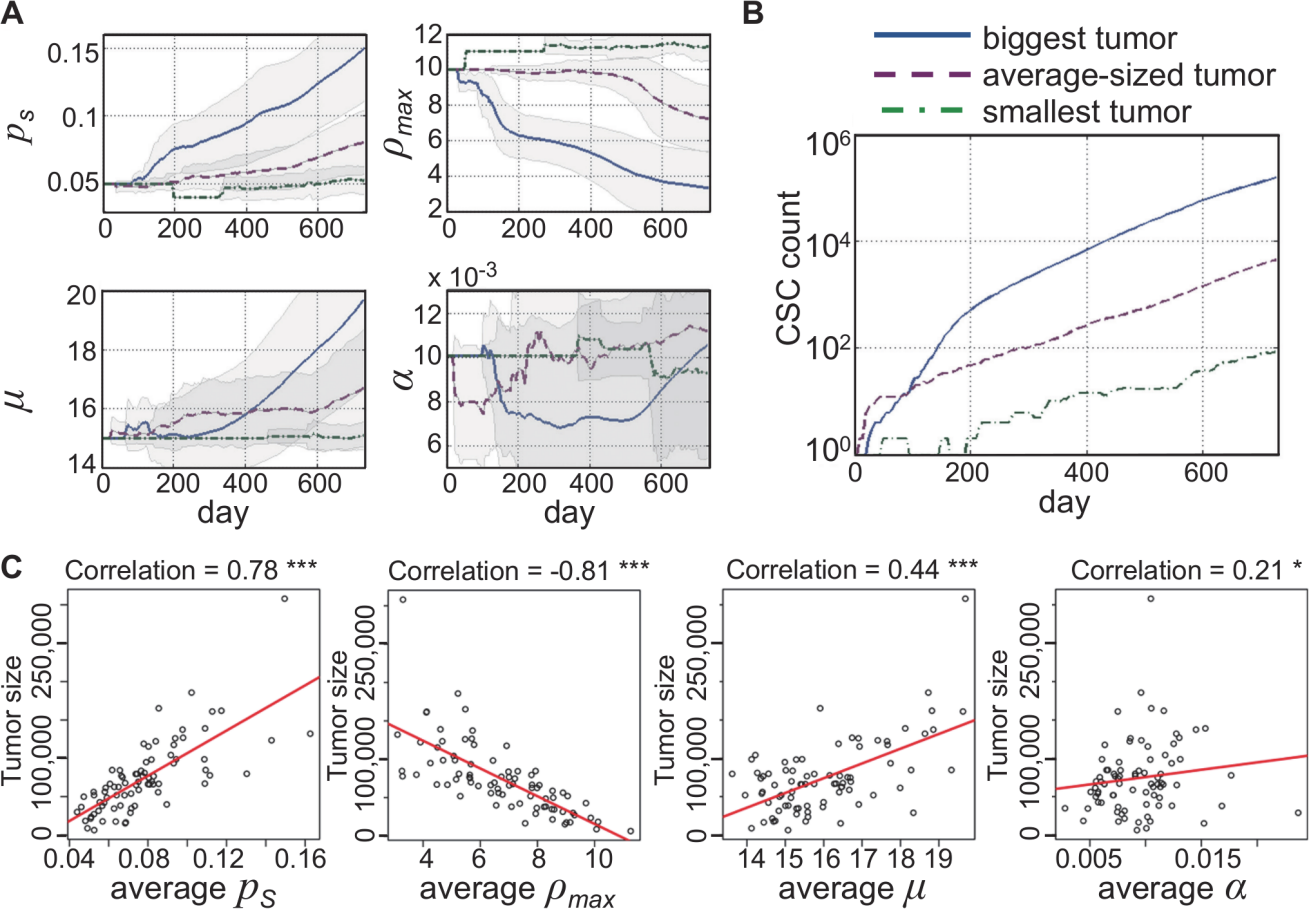
A) Evolution of cancer stem cell traits in the biggest (solid blue curve), an average-sized (red dashed) and the smallest tumor (green dot-dashed). p_s_: probability of symmetric division; ρ_max_: proliferation potential; μ: migration rate, α: spontaneous cell death probability. **B)** Cancer stem cell count over time in the biggest (solid blue curve), an average-sized (red dashed) and the smallest tumor (green dot-dashed). **C)** Correlation of tumor size with each average trait parameter. ***p<0.001; *p<0.05.

We now investigate the CSC trait vectors that dominate the smallest, an average-sized and the largest evolved tumor to identify favorable and unfavorable evolutionary trajectories. Fig. 1C-E shows the temporal evolution of the most common trait vectors and parameter values for the 10 dominant vectors at the end of the simulation (t = 730). Only 28 phenotypes have evolved in the smallest tumor after 730 days. Such small pool of available phenotypes indicates that early unfavorable mutations blocked the ability of the tumor to develop a wide spectrum of phenotypes. The largest tumor, in contrast, evolved more than 11,000 distinct phenotypes. The 10 most common phenotypes reveal that the biggest evolutionary change manifested itself in decreased proliferation capacity ρ_max_. Indeed, the most common phenotypes in the smallest tumor have increased value of proliferation capacity suggesting competition between CSCs and CCs and long population level dormancy periods as previously described [16,30]. The important role of CC proliferation potential in tumor is further emphasized in detailed analysis of the individual trait evolution and resulting CSC numbers (Fig. 2A). An early decrease in proliferation potential ρ_max_ allows for increased cancer stem cell proliferation and subsequent trait mutation events. An increase in symmetric division probability p_s_ and a decrease in cell death α, and a later increase in cell migration follow the initial decrease in ρ_max_. An early increase in proliferation potential ρ_max_ inhibits cancer stem cell pool expansion and diminishes further trait evolution (Fig. 2B). Correlation analysis further supports the pivotal role of CC proliferation potential in determining total tumor size. Symmetric division probability p_s_ and cell migration μ correlate positively with tumor size. A weak but significant positive correlation is also observed with spontaneous cell death α (Fig. 2C). This lends support to previous theoretical observations that increased cell death counteracts cancer stem cell confinement [16,31] and promotes self-metastatic tumor expansion [27].

### Spatial phenotypic heterogeneity and implications for tumor biopsy

**Fig 3:**
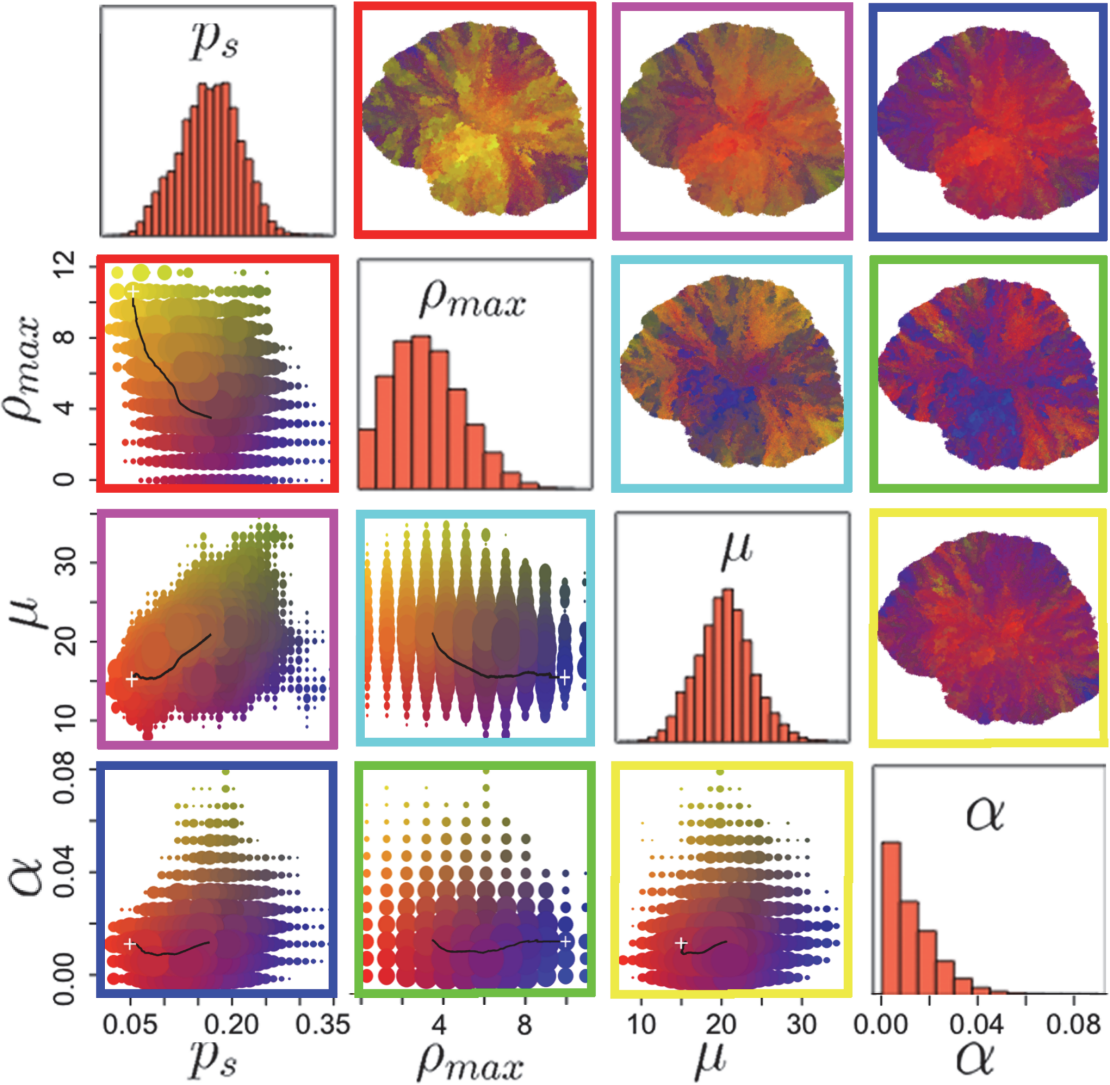
Parameter distribution in the biggest evolved tumor at t = 730 days. Plots below the diagonal show the parameter pair prevalence in the tumor as well as the evolutionary trajectory of the parameter pair (+ marks the initial condition). Plots above the diagonal show the color-coded morphological distribution of parameter pairs in the final tumor. Colors as in corresponding plots below the diagonal.

Evolutionary phenotypic heterogeneity leads to unpredictable spatiotemporal distribution of cells with varying aggressiveness. Whilst some tumor regions can be dominated by an ancestral phenotype, more recently evolved clones could be spatially limited to small pockets albeit large fitness. However, similar phenotypes may also develop along independent evolutionary trajectories at different spatial locations throughout the tumor. Cells with phenotypes close to the initial trait vector are contained in the tumor core. More recently evolved clones with increasing aggressiveness are located at the tumor periphery where they dominate outgrowth in circular manner (Fig. 3). Throughout the tumor, the probabilities of symmetric CSC division as well as migration rate have a monotonically increasing average value and follow a bell shaped distribution at t = 730 days. Proliferation potential and cell death distributions are skewed to the left approaching zero (Fig. 3).

**Fig 4:**
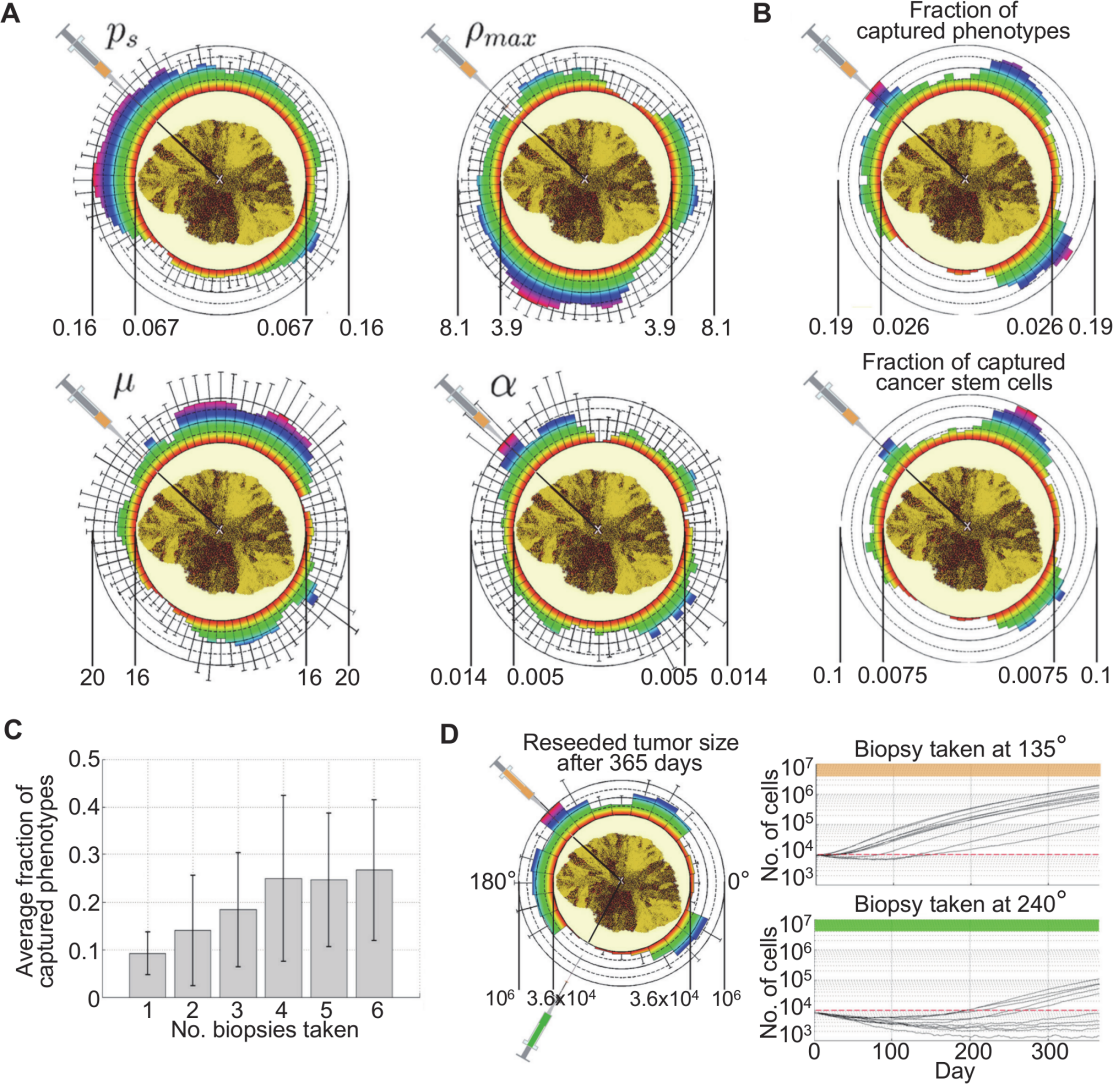
A) Distribution of average parameter values and standard deviations in biopsy samples taken at 5-degree intervals around the tumor. Color-coded heat map to further emphasize variation. p_s_: probability of symmetric division; ρ_max_: proliferation potential; μ: migration rate, α: spontaneous cell death probability. **B)** Fraction of captured phenotypes (ratio of unique phenotypes in a sample to the total number of unique phenotypes in the whole tumor) and fraction of cancer stem cells (ratio of CSC count to the total cell count within the collected sample) in biopsy samples taken at 5-degree intervals around the tumor. **C)** Average fraction of phenotypes captured with multiple equally distant biopsies within a 90-degrees tumor quadrant. **D)** Different tumor growth dynamics of re-seeded subpopulations from biopsy samples (10 subpopulations, ~10,000 cells each) taken at 135° (orange) and 240° (green).

We simulate *in silico* biopsy on the largest evolved tumor to investigate phenotypic diversity in the biopsy sample. A 26 Gauge biopsy needle with a 0.46 mm diameter is simulated to penetrate the tumor from the boundary to the center of mass. All cells (~100,000) in the needle trajectory are collected in a single harvest and their phenotypic traits averaged. Biopsies are collected at different angles in 5 degrees intervals yielding 72 samples. The average value for different phenotypic traits can vary orders of magnitude dependent on biopsy angle (Fig. 4A), suggesting spatially localized evolution and diverse phenotypic dispersal. Whilst the fraction of captured phenotypes (ratio of unique phenotypes in a sample to the total number of unique phenotypes in the whole tumor) varies by one order of magnitude, the fraction of cancer stem cells in the collected samples (ratio of CSC count to the total cell count within the collected sample) vary by as much as two orders of magnitude ranging from 0.7% to 10% (Fig. 4B). Multiple equally distant biopsies within a 90-degrees tumor quadrant increase the average fraction of captured phenotypes from 9% for one biopsy to 25% for 4 or more biopsies (Fig. 4C). Single collected biopsy samples can be divided into multiple subpopulations (10 subpopulations with approx. 10,000 cells each in our settings) and reseeded to obtain sample growth dynamics. Biopsy-specific phenotypic diversity yields tumor populations with grossly different growth dynamics from rapid and persistent growth (for biopsy taken at 135°) to initial decay and long-term dormancy (240°; Fig. 4D).

## Discussion

It becomes increasingly appreciated that tumors are heterogeneous populations of cells with different traits and fates. An intrinsic difference in tumor initiation and propagation potential led to a classification of cancer stem cells (CSC) and non-stem cancer cells (CC) in the majority of hematologic and solid tumors [12,32–34]. The transformed cells that lead to the initial CSC inherit kinetic properties of their somatic cell of origin, which may be significantly varying at different ages and between organs. Different cell kinetics result in different overall tumor population dynamics ranging from population level dormancy to aggressive growth and invasion [17]. Agent-based models are well suited to simulate individual cell dynamics and estimate tumor progression dependencies on single cell kinetics. We have identified a set of orthogonal cell kinetics that include cell migration rate, proliferation potential, spontaneous cell death, and symmetric cancer stem cell division. In a cancer stem cell-driven tumor, proliferation potential and spontaneous cell death have been shown to non-monotonically modulate overall tumor progression [16,30,31] while increased cell migration and symmetric CSC division always lead to accelerated tumor growth [27,35]. The complex interplay of these participating dynamics, however, requires a more elegant investigation of which combinations of simultaneously evolving kinetics yield the most aggressive tumor clones. While parameter evolution yields predominantly fast growing tumors, early unfavorable mutation events may ultimately push tumor dynamics into dwarfed tumor growth or long-term dormancy. Our study suggests that cell proliferation potential is the strongest modulator of tumor growth. Early increase in proliferation potential yields larger populations of CC that compete with CSC and thus inhibit CSC division [16,30]. Conversely, a reduction in proliferation potential decreases intratumoral competition and enables accelerated CSC pool expansion and further evolution. Interestingly, short telomeres indicative of short-lived cancer cells with limited proliferation capacity have indeed been observed in malignant tumor population [26,36], lending further support that limiting the number of CC progeny promotes parental CSC expansion and tumor growth. Phenotypes that eventually dominate the tumor developed relatively late indicating that the chosen initial parameter values were suboptimal for tumor progression despite previous observations of relatively fast growth [17]. Successfully evolving tumors exhibit a heterogeneous distribution of phenotypes with different sub-clones dominating local expansion. This confirms clinical observations of intratumoral heterogeneity and branched evolution with spatially distinct genetic profiles [37]. The spatial heterogeneity of cell traits also leads to a large variation in CSC fraction in biopsy samples, offering yet another angle to the ongoing discussion about the proportion of tumorigenic subpopulations in tumors [38–41]. Furthermore, our work suggests that the so-called sub-population of cancer stem cells is in itself heterogeneous. Whilst some CSC will initiate immediate growth, other CSC will form microscopic tumors that may remain dormant for prolonged periods of time. Indeed, a variety of individual human derived malignant cancer cell lines can each be carefully divided into sub-clones that form fast-growing tumors or stable disease for many months before initiating rapid growth [42–44].

The model presented here is a complex but simple approach for measuring continuous intratumoral CSC evolution. For computational convenience we have limited our study to a two-dimensional model. A previous analysis in a similar cell interaction model has shown that two-dimensional cellular automata qualitatively mimic three-dimensional tumor growth dynamics [45]. The presented results assumed a mutation rate of 50%; other mutation rates yield qualitatively similar results on a different time scale with smaller mutation rates yielding slower growing tumors (S1 Fig). To first understand the trajectories of intratumoral evolution, we limited the study to stem and non-stem cancer cells and ignored tumor environmental factors that will exert external selection pressure on the tumor population and shape parameter fitness values accordingly [46,47]. Future developments of this model may include local tumor-environmental interactions or globally-informed modulations by the host immune system. To further increase complexity, bi-directional plasticity of phenotypes via CSC differentiation and CC de-differentiation may be considered [48–51], which will contribute to increased biological realism of tumor evolution dynamics.

## Methods

**Fig 5:**
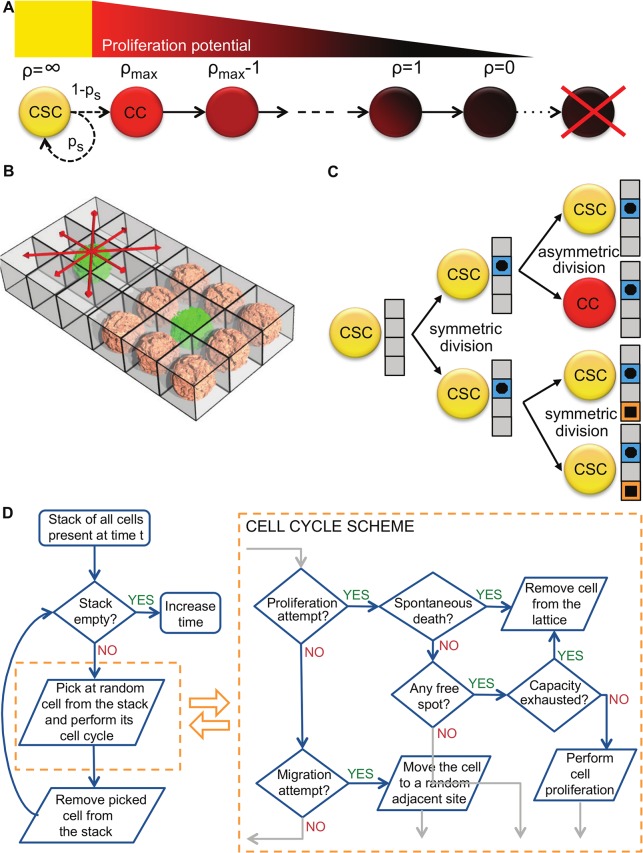
A) Schematic of the cellular hierarchy and proliferation potential attrition in the non-stem cancer cell population. **B)** Schematic representation of competition for space in a regular lattice. **C)** Schematic of the division-dependent trait evolution. Only during symmetric CSC divisions traits are subject to evolution. **D)** Schematic of the simulation procedure and cell cycle evaluations.

We develop a theoretical framework to investigate tumor progression in response to cancer stem cell evolution. We explore orthogonal cell traits including cell migration to facilitate invasion [52], spontaneous cell death due to genetic drift after accumulation of irreversible deleterious mutations [53,54], symmetric cancer stem cell division that increases the cancer stem cell pool [55,56], and telomere length and erosion as a mitotic counter for inherited non-stem cancer cell proliferation potential [22,57]. We use an agent-based model to simulate the dynamics of single cells and observe cell-cell interactions and population-level tumor formation [16,27]. The model is realized as an asynchronous cellular automaton in which cell events are stochastically driven. A cell, either cancer stem cell (CSC) or non-stem cancer cell (CC), occupies a single grid point of (10μm)^2^ on a two-dimensional square lattice. Each CSC is characterized by its specific trait vector [p_s,_ ρ_max_, μ, α] denoting probability of symmetric division, proliferation capacity, migration potential and spontaneous death probability, respectively. According to the cancer stem cell hypothesis, CSCs have unlimited proliferation potential and thus their proliferative capacity ρ_max_ does not exhaust. At each division CSCs produce either another CSC with probability p_s_ (symmetric division) or a CC with probability 1-p_s_ (asymmetric division). CCs that are direct offspring of a CSC inherit the initial proliferation capacity ρ that decreases with each cell division (Fig. 5A). At ρ = 0, CCs die and are removed from the simulation. At each proliferation attempt, cells may undergo spontaneous death with probability α and then be removed from the system. Both tumor subpopulations are equipped with migration potential μ representing number of potential cell displacements into neighboring lattice sites per day. We assume that cells need adjacent space for migration and proliferation, and cells that are completely surrounded by other cells (eight on a two-dimensional lattice) become quiescent (Fig. 5B). In unsaturated environments, cells proliferate and migrate into vacant adjacent space at random. To avoid artifacts caused by computational domain boundaries we introduce a dynamically growing domain [58].

Motivated by the immortal strand hypothesis [59] and non-random DNA strand co-segregation [60] we model mutation events in the malignant subpopulation of CSCs during symmetric division (Fig. 5C). We ignore mutation of traits in CCs as those will be lost from the total population [61]. Traits are marginally mutated allowing for continuous evolution and large phenotypic variety, assuring that the phenotype pool is not limited to a number of trait combinations from which a phenotype is randomly drawn [35,62,63]. We assume that a single mutation affects at most one trait and induces a stochastic positive or negative unit change of the trait parameter value, i.e. p_s_±0.01, ρ_max_±1, μ±1, or α±0.001. The trait to be mutated is chosen at random from a discrete uniform distribution. The modified trait vector is inherited by both CSCs and then further propagated to their respective CC populations. If a trait becomes negative the cell is considered unviable and removed from the simulation. Fig. 5D summarizes the simulation process for the presented model.

Simulation time is advanced at discrete time intervals Δt = 1/24 day (i.e., 1 hour), that is 24 simulation steps equal one day. At each simulation time step, cells are considered in random order and the behavior of each cell is updated. Cell proliferation, migration and death are random events with the respective probabilities scaled to simulation time. Cell proliferation and migration are temporally mutually exclusive events, and cell death only occurs when cell actively attempts to proliferate. We assume that cells proliferate on average once per day (proliferation probability p_d_ = 1×Δt), migrate with probability (1-p_d_)p_m_ and die with probability p_d_α. Let p_m_ = μ×Δt, where the parameter μ denote motility of cancer cells. Due to the stochastic nature of the model we perform at least 100 independent simulations for each discussed case and report average values and standard deviations.

## Supporting Information

S1 Fig Comparison of total cell count, stem cell count and stem cell fraction over time for tumors with different probability of mutation p_mut_. B) Evolution of cancer stem cell traits in the biggest (solid blue curve), an average-sized (red dashed) and the smallest tumor (green dot-dashed) evolved with mutation probability p_mut_ = 0.1 (10%). p_s_: probability of symmetric division; ρ_max_: proliferation potential; μ: migration rate, α: spontaneous cell death probability. C) Cancer stem cell count over time in the biggest (solid blue curve), an average-sized (red dashed) and the smallest tumor (green dot-dashed) evolved with mutation probability p_mut_ = 0.1. C) Correlation of tumor size with each average trait parameter for tumors evolved with mutation probability p_mut_ = 0.1. ***p<0.001; *p<0.05.
(TIF)
