# Supplementary figures and images for: Evolution and Phenotypic Selection of Cancer Stem Cells
Source: PLoS Comput Biol. 2015 Mar 5;11(3):e1004025. doi: 10.1371/journal.pcbi.1004025 (PMC4351192; doi:10.1371/journal.pcbi.1004025)

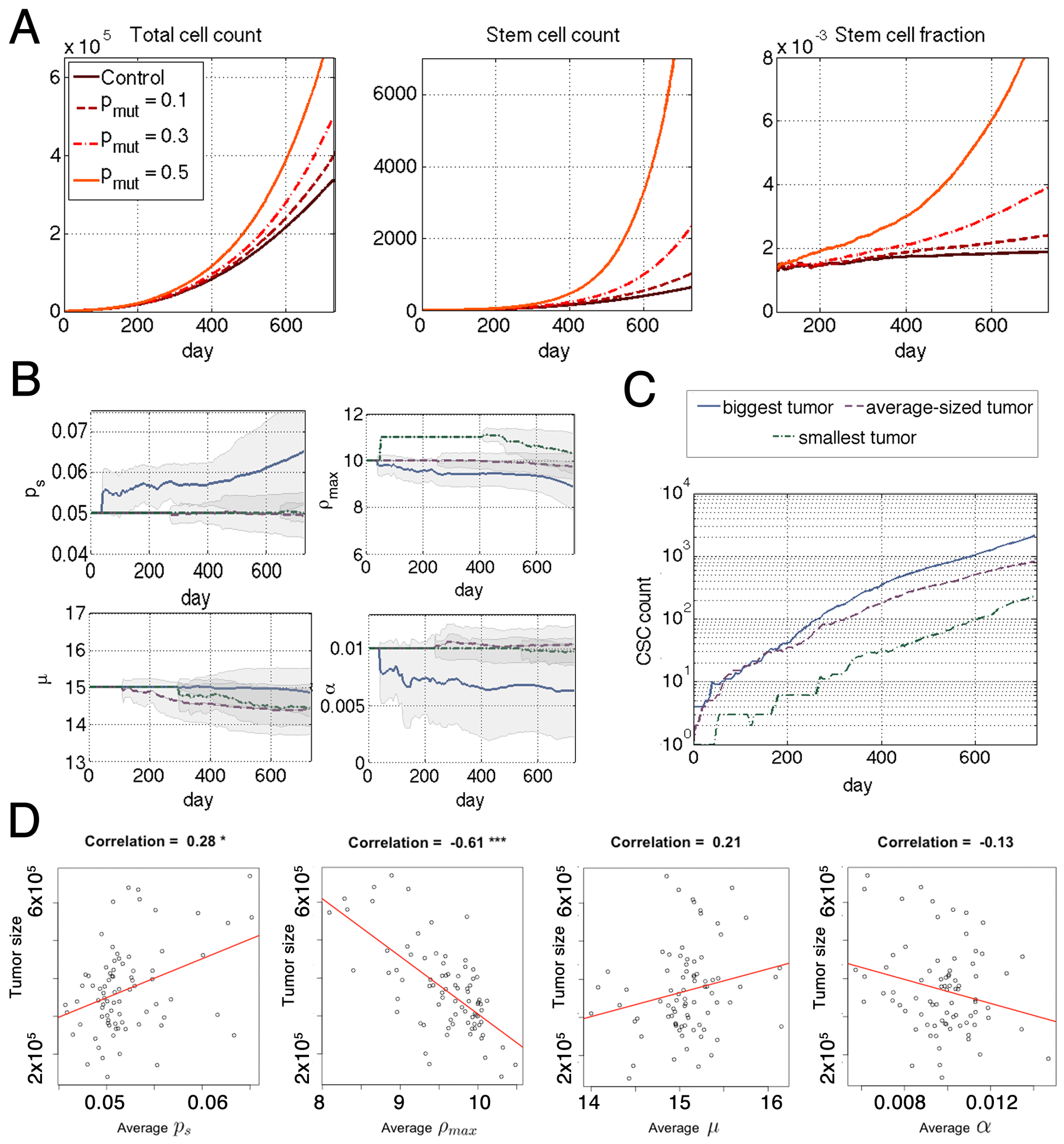

Supplement: S1 Fig — B) Evolution of cancer stem cell traits in the biggest (solid blue curve), an average-sized (red dashed) and the smallest tumor (green dot-dashed) evolved with mutation probability pmut = 0.1 (10%). ps: probability of symmetric division; ρmax: proliferation potential; μ: migration rate, α: spontaneous cell death probability. C) Cancer stem cell count over time in the biggest (solid blue curve), an average-sized (red dashed) and the smallest tumor (green dot-dashed) evolved with mutation probability pmut = 0.1. C) Correlation of tumor size with each average trait parameter for tumors evolved with mutation probability pmut = 0.1. ***p<0.001; *p<0.05. (TIF) [file pcbi.1004025.s001.tif]
